# Research of Mitochondrial Function, Structure, Dynamics and Intracellular Organization

**DOI:** 10.3390/ijms24010886

**Published:** 2023-01-03

**Authors:** Andrey V. Kuznetsov, Michael J. Ausserlechner

**Affiliations:** Department of Pediatrics I, Medical University of Innsbruck, A-6020 Innsbruck, Austria

Mitochondria have been recognized as the energy (in the form of ATP)-producing cell organelles, required for cell viability, survival and normal cell function. However, mitochondria perform several other important cellular functions, participating in many cellular metabolic processes [1,2,3]. They regulate ROS and Ca^2+^ signaling and cell redox states. In addition, mitochondria are also involved in the induction of apoptosis as well as autophagy. Mitochondria participate in the metabolism of steroid hormones, in urea, lipid and amino acids cycles. In addition, they play crucial roles in glucose and insulin regulations [4]. Importantly, mitochondria can sense their intracellular environment, as cellular O_2_, Ca^2+^, ROS and ATP levels and the presence/absence of growth factors [5,6]. The existence of local functional enzyme channeling and coupling may result in significant mitochondrial heterogeneity, also leading to the existence of various mitochondrial subpopulations with different properties. In addition to their role in cellular bioenergetics, changes in the complex mitochondrial metabolism, their permeability and dynamics are crucial in the cells’ fates, decisions and injury. However, the exact interplay between all these functions is not yet known. Many studies performed in various laboratories have demonstrated a strict interconnection between mitochondrial and entire cell physiology [7]. The mitochondrial respiratory activities coupled with the electron transfer are considered as a major source of cellular reactive oxygen species (ROS) generation, leading to oxidative stress [8,9]. A central role of mitochondrial changes/damages has been demonstrated to be associated with impairment of cellular metabolism/viability in numerous injuries and diseases. Mitochondrial involvement has been shown in various genetic diseases, congestive heart failure (CHF), ischemia/reperfusion injury (IRI), various myopathies, neurodegenerative diseases, diabetes, cancer and obesity. Interest in mitochondria was considerably reintroduced after the discovery of their critical role in the induction of apoptosis, by the release of various pro-apoptotic factors, such as cytochrome c, AIF, etc. [10,11,12]. Therefore, several factors and signals from mitochondria may influence the cell metabolism (signaling out). The interaction of mitochondria with various cytoskeletal proteins such as beta-tubulin and specific isoforms of plectin can dynamically participate in the regulation of mitochondrial function via the outer membrane protein VDAC (Voltage Dependent Anion Channel) [13,14]. Mitochondria-endoplasmic reticulum (ER) interactions have been observed to be crucial for Ca^2+^ homeostasis [15]. Nevertheless, several concerns and questions remain and need further in-depth analysis.

The intention of the review by Kuznetsov et al. was to present and highpoint several potent approaches and most frequently used methods/techniques for the analysis of mitochondrial functions, structures, and organization, in cardiac and skeletal muscle cells in situ. The authors noted that the main metabolic and functional characteristics of mitochondria obtained in situ (in permeabilized cells and tissue samples) and in vitro (in isolated mitochondria) are quite different, which challenges interpretations of experimental and clinical data. These differences are explained by the existence of the mitochondrial network that possesses multiple interactions with the cytoplasm and other subcellular organelles and proteins. Metabolic and functional crosstalk between mitochondria and other subcellular organelles (cytoskeleton and ER) plays a crucial role in the regulation of mitochondrial metabolism and physiology. Therefore, it is important to analyze mitochondria in vivo or in situ without their isolation from the natural cellular environment. The authors emphasize that the combination of high-resolution respirometry and fluorescent confocal microscopy has advantages. Fluorescent imaging of mitochondria allows, not only to analyze mitochondrial organization and structure, but also to monitor mitochondrial dynamics (fission, fusion, motility). Moreover, using internal auto-fluorescence of NADH and flavoproteins, mitochondrial imaging can analyze mitochondrial function. The application of various fluorescent probes provides the possibility to analyze mitochondrial membrane potential, ROS and calcium. This review summarizes previous studies and discusses existing approaches and methods for the analysis of mitochondrial function, structure, and intracellular organization in situ [16].

In the review by A. Faria-Pereira and V.A. Morais, the authors discuss the two pathways for energy production through glycolysis and mitochondrial oxidative phosphorylation, particularly focusing on the balance of glycolysis and oxidative phosphorylation to meet energy demands in both synaptic and other brain mitochondria. The authors emphasize that besides energy needs, synaptic mitochondria are also important for ROS regulation and calcium homeostasis, whereas dysfunction of glycolytic and mitochondrial bioenergetics is associated with synaptic energy deficits neurodegenerative diseases and synapse degeneration [17].

The paper by Shuchan Sun et al. presented a new form of puerarin, puerarin-V and investigated its role for cardioprotection in the diabetic cardiomyopathy (DCM). The authors tried to investigate possible mechanisms of puerarin-V beneficious action in the pathophysiology of DCM. Moreover, the effects of puerarin-V were compared with other beneficial agents such as metformin and API, showing that puerarin-V can better reduce myocardial damage in DCM rats. The authors concluded that this study may provide a new therapeutic approach for the clinical treatment of DCM [18].

The article by Filipe Cortes-Figueiredo et al. presents new, innovative methods for the sequencing and data analysis of mitochondrial DNA (mtDNA). The authors indicate Applied Biosystems™ Precision ID mtDNA Whole Genome Panel (Thermo Fisher Scientific, USA) is an innovative library preparation kit suitable for degraded samples and at low DNA. The authors present an alternative customizable pipeline, the PrecisionCallerPipeline (PCP), for processing samples with the correct rCRS output after Ion Torrent sequencing with the Precision ID library kit [19].

In the article by T. Marutani et al., the authors present novel, mitochondria-based (produced from mitochondrial cytochrome b) neutrophil-activating peptide—Mitocryptide-2 (MCT-2). The authors suggest that MCT-2 can represent a novel important factor that not only initiates innate immunity via the specific activation of factor (FPR2), but also promotes delayed responses by the activation of factor FPR1, which may include resolution and tissue regeneration. The results of this paper point to the necessity of the exact structures analysis of activating factors for the investigation of intrinsic immune responses [20].

In the article by S. Nesterov et al., the authors applied sub-tomogram averaging in situ and cryo-electron tomography to confirm structurally functional data, suggesting the existence of mitochondrial inner membrane super-complexes consisting of electron transport (respiration) system and ATP synthase. Using their approaches, the authors show structural link between respiratory chain and ATP synthases super-complexes that form ordered clusters, demonstrating thus a new type of structural organization of mitochondrial oxidative phosphorylation. These super-complexes may provide an advantage for more quick and efficient communication between respiratory chain and ATP synthase [21].

In the review by P. Kowalczyk et al., the authors summarize and discussed the most important experimental findings that show how mitochondrial oxidative stress is associated with a wide variety of diseases and injuries, including Alzheimer disease (AD), autoimmune type 1 diabetes and several others. It is well known that oxidative stress and ROS produce deleterious effects on cellular biomolecules, including proteins, biomembranes and DNA. In this review, several attempts to reduce oxidative stress as a therapeutic approach are also discussed [22].

In the article by Ah-Ra Lyu et al., the authors have demonstrated that diabetes in mice models was associated with hearing loss, due to a reduction in cochlear blood flow and expression of C-terminal-binding protein 2 (synaptic marker). This hearing loss was associated also with significant damage of mitochondria in cochlea and a reduction of mitochondrial cytochrome c oxidase (respiratory complex IV) function. The authors concluded that mechanisms of diabetic hearing loss involve synapses injury, damage to mitochondria and activation of apoptosis. Therefore, the authors suggested the benefits of mitochondrial targeting as a possible strategy for therapies [23].
